# Phosphatidic acid produced by phospholipase Dα1 and Dδ is incorporated into the internal membranes but not involved in the gene expression of *RD29A* in the abscisic acid signaling network in *Arabidopsis thaliana*

**DOI:** 10.3389/fpls.2024.1356699

**Published:** 2024-04-12

**Authors:** Ruth Ndathe, Naohiro Kato

**Affiliations:** Department of Biological Sciences, Louisiana State University, Baton Rouge, LA, United States

**Keywords:** ABA, signaling network, phosphatidic acid, PLD, RD29A, ABRE

## Abstract

Core protein components of the abscisic acid (ABA) signaling network, pyrabactin resistance (PYR), protein phosphatases 2C (PP2C), and SNF1-related protein kinase 2 (SnRK2) are involved in the regulation of stomatal closure and gene expression downstream responses in *Arabidopsis thaliana*. Phosphatidic acid (PA) produced by the phospholipases Dα1 and Dδ (PLDs) in the plasma membrane has been identified as a necessary molecule in ABA-inducible stomatal closure. On the other hand, the involvement of PA in ABA-inducible gene expression has been suggested but remains a question. In this study, the involvement of PA in the ABA-inducible gene expression was examined in the model plant *Arabidopsis thaliana* and the canonical *RD29A* ABA-inducible gene that possesses a single ABA–responsive element (ABRE) in the promoter. The promoter activity and accumulation of the *RD29A* mRNA during ABA exposure to the plants were analyzed under conditions in which the production of PA by PLDs is abrogated through chemical and genetic modification. Changes in the subcellular localization of PA during the signal transduction were analyzed with confocal microscopy. The results obtained in this study suggest that inhibition of PA production by the PLDs does not affect the promoter activity of *RD29A*. PA produced by the PLDs and exogenously added PA in the plasma membrane are effectively incorporated into internal membranes to transduce the signal. However, exogenously added PA induces stomatal closure but not *RD29A* expression. This is because PA produced by the PLDs most likely inhibits the activity of not all but only the selected PP2C family members, the negative regulators of the *RD29A* promoter. This finding underscores the necessity for experimental verifications to adapt previous knowledge into a signaling network model before its construction.

## Introduction

Abscisic acid (ABA) is an important plant hormone that regulates plant signaling networks, such as seed maturation and dormancy, and mediates the response of plants to abiotic stresses such as drought, cold, freezing, and salinity (Sondheimer et al., 1968); (Finkelstein et al., 1985); (Mantyla et al., 1995); (Borovskii et al., 2002). ABA-mediated responses to drought stress and salinity include regulation of stomatal closure (Kriedemann et al., 1972); (Jackson and Hall, 1987; Steuer et al., 1988) and altered gene expression (Gómez et al., 1988); (Mundy and Chua, 1988). These two responses are distinct in the time of response. While stomatal closure takes minutes, expression of most of the ABA-responding genes reaches a plateau after 8 hours of ABA exposure (Song et al., 2016). The fast response in the stomata occurs due to protein phosphorylation, which leads to the activation of anion channels and subsequent efflux of ions and water out of the guard cells, leading to loss of turgor-inducing stomatal closure (Schmidt et al., 1995) (Pei et al., 1997). The slow response in ABA-induced gene expressions such as *RD29A*, *RD29B*, and *RAB18* occurs due to the modification of transcription factors by phosphorylation, which leads to the activation of the respective promoters (Xiong et al., 2002; Zhu, 2002).

These two responses share the core components of the ABA signaling pathway (**Supplementary Figure S1**), which starts with the binding of ABA to pyrabactin resistance/pyr1-like/regulatory components of ABA receptors (PYR/PYL/RCAR) (Melcher et al., 2009; Park et al., 2009; Santiago et al., 2009). This causes the binding of PYR to protein phosphatases 2Cs (PP2Cs) (Santiago et al., 2009; Soon et al., 2012). Without PYR binding, PP2Cs bind and dephosphorylate SNF1-related protein kinase 2 (SnRK2) (Umezawa et al., 2009; Yoshida et al., 2010). Therefore, in the presence of ABA, SnRK2 is free from PP2C and phosphorylates downstream signaling proteins. In stomatal closure, the activated SnRK2.6/OST1 (open stomata 1) phosphorylates the slow anion channel 1 (SLAC1) that upregulates ion uptake (Geiger et al., 2009; Lee et al., 2009). The SnRK2.6 and additional kinases SnRK2.2 and SnRK2.3 phosphorylate ABRE-binding factors (ABFs), which are transcription factors that bind ABA-responsive elements (ABREs) on ABA-induced genes (Johnson et al., 2002; Furihata et al., 2006). Sharing of the core components by both responses, i.e., stomatal closure and gene expression, is most evident in genetic analyses. For instance, *pyr* quadruple mutant (*pyr1*/*pyl1*/*pyl2*/*pyl4*) plants are impaired in ABA-induced stomatal closure and ABA-induced gene expression (Park et al., 2009; Nishimura et al., 2010). On the other hand, *pp2c* triple mutant (*hab1-1*/*abi1-2/abi2-2*) plants are hypersensitive in both ABA-induced stomatal closure and ABA-induced gene expression (Rubio et al., 2009), while *snrk2* triple mutant (*snrk2.2*/*2.3*/*2.6*) plants are impaired in both ABA-induced stomatal closure and ABA-induced gene expression (Lin et al., 2021).

The involvement of phosphatidic acid (PA) in the ABA signaling network concerning stomatal closure has been demonstrated (Zhang et al., 2004) (**Supplementary Figure S1**). PA is a membrane component but also functions in various aspects of plant physiology, such as abiotic stress response, polarized cell growth, and cytoskeletal changes (Frank et al., 2000; Munnik et al., 2000; Lee et al., 2003; Wang et al., 2019; Lee et al., 2003; Kolesnikov et al., 2022). It is known that PA is transiently formed in the plasma membrane by phospholipase Ds (PLDs) within the first 10 minutes after plants are exposed to ABA (Ritchie & Gilroy, 1998; Zhang et al., 2004). PA in the plasma membrane then binds ABA insensitive 1 (ABI1), a PP2C phosphatase, inhibiting its phosphatase activity and tethering it to the plasma membrane, which makes the SnRK2 kinases freely mobilized within the cells to activate SLAC1 anion channels in stomatal closure (Ritchie and Gilroy, 1998; Zhang et al., 2004; Mishra et al., 2006). The formation of PA also stimulates ABA-induced ROS production by binding and activating NADPH oxidase, RbohD/F, in the guard cells to promote stomatal closure (Zhang et al., 2009). Further regulation involves PLDα1 protein, which binds the heterotrimeric G protein subunit Gα, limiting ABA-mediated inhibition of the stomatal opening (Zhao and Wang, 2004). Involvement of PA in the ABA signaling network has been clearly shown genetically in *pld* mutant plants (*pldα, pldδ*, and *pldα1/δ*). Single mutants *pldα1* and *pldδ* plants were shown to be impaired in ABA-mediated stomatal closure (Mishra et al., 2006; Zhang et al., 2009; Distéfano et al., 2012; Zhang et al., 2004; Guo et al., 2012a) similar to the *pldα1/δ* double knock out mutants in (Uraji et al., 2012). In addition, the exogenous application of PA induces stomatal closure in both wild-type and the mutant plants (Mishra et al., 2006; Uraji et al., 2012; Distéfano et al., 2012).

The involvement of PA formed by the PLDs in downstream ABA-mediated gene expression has also been suggested, but the evidence contradicted each other (**Supplementary Figure S1**). Previous studies have investigated this using knockout mutants and overexpression of the *PLD* genes. *PLDα1* gene overexpression led to enhanced expression of both *RD29A* and *RAB18* in dehydration (Peng et al., 2010). On the other hand, a study on *PLDδ* gene knockout mutant found that ABA upregulates expression levels of *RD29A* and *RAB18* (Distéfano et al., 2015). In addition, in a study on the effect of PA on downstream ABA-mediated gene expression using a PLD chemical inhibitor, 1-butanol, no effect on sodium chloride-mediated *RD29A* expression was observed (Thiery et al., 2004). In contrast, *RAB18* expression was down-regulated. Furthermore, ABA-stimulated PLD activates anion channels, leading to the *RAB18* gene expression in Arabidopsis suspension cells (Hallouin et al., 2002).

Another discrepancy is the subcellular localization of the ABI1 and PLDα1 proteins. As described above, tethering ABI1 in the plasma membrane was thought to promote ABA signaling (Zhang et al., 2004). However, recent studies have shown that ABI1 localizes in the cytosol, the nucleus, and the plasma membrane. Nucleic localization was essential to enable its inhibitory role in ABA-induced gene expression (Moes et al., 2008; Umezawa et al., 2009); (Li et al., 2012). Subcellular localization of the PLDα1 was also found in the plasma membrane, microtubules, and cytosol (Li et al., 2009).

We previously constructed a dynamic model of ABA-inducible expression of the Arabidopsis *RD29A* (Ndathe et al., 2022). Because the promoter of *RD29A* contains a single ABRE cis-element (Yamaguchi-Shinozaki and Shinozaki, 1994), the expression of *RD29A* has been most widely used as a marker for activating core components of the ABA signaling pathway in the past (Nakashima et al., 2006; Zhu et al., 2017). Indeed, 53% of a total of 6,514 genes in the ABA gene regulatory networks contain a single ABRE (Ndathe et al., 2022; Sun et al., 2022). The expression is determined by detecting the accumulated mRNA in cells (Narusaka et al., 2003; Lee et al., 2016) or by detecting the activity of the recombinant protein, such as luciferase (LUC) under the *RD29A* promoter (Yamaguchi-Shinozaki and Shinozaki, 1993; Ishitani et al., 1997; Msanne et al., 2011). The model analysis followed by experimental verification found that exposing ABA is sufficient to express *RD29A*, but simultaneous binding of dehydration-responsive element binding protein 2A (DREB2A) and ABF to the promoter is required (**Supplementary Figure S1**). In this case, PP2C feedback controls the time scale and dynamic expression range in genes containing the single ABRE in the promoter (Ndathe et al., 2022).

In this study, we investigated the involvement of PA in *RD29A* expression to expand the construction of the dynamic model. Because stomatal closure and gene expression share the core protein components of the ABA-signaling network, it is plausible that PA is also involved in ABA-inducible gene expression. To examine PLD activity and PA localization during the ABA signal transduction, we took not only a genetic approach but also a cell biology approach with fluorescently labeled PA and phosphatidylcholine (PC) that have been used in plants (Yamaguchi et al., 2004; Zhang et al., 2004; Guo et al., 2012b).

Here, we show that PA produced by PLDs, and exogenously added PA in the plasma membrane is effectively incorporated into the internal membranes to transduce the signal. However, exogenously added PA induces stomatal closure but not *RD29A* expression.

## Materials and methods

### Plant growth conditions

*Arabidopsis thaliana* seeds were obtained from Arabidopsis Biological Resource Center (ABRC). The pldα1/δ double knockout mutant seeds were obtained from the laboratory of Dr. Xuemin Wang. The seeds were sterilized in 70% Ethanol for 1 minute, 50% bleach, and 0.05% Triton-X100 for 10 minutes. The seeds were then rinsed 6 times with sterile distilled water and plated on 0.8% agar ½ MS strength. They were then stratified at 4 ° C for 3 days, then grown in a growth chamber under a growth cycle of 16 hours light, 8 hours dark with a light set at 100 µmol m^-2^ s^-1,^and 22 ° C.

### *RD29A::LUC* expression assay

One-week-old seedlings with *RD29A::LUC* (Col) (CS67900) or *CAMV35S::LUC* (Col) (CS25237) and (CS25230) were placed in wells of a 96-well plate (Thermo Fisher cat# 267350) and incubated with either 200 µM ABA only or 200 µM ABA with 1 µM 5-Fluoro-2-indolyl des-chlorohalopemide (FIPI) (Millipore Sigma cat# 528245) or with 100 µM egg yolk PA (Avanti Polar lipids cat# 840101). For control, equal amounts of DMSO were used. Three seedlings were placed in one well for *RD29A::LUC*, and one seedling for *CAMV35S::LUC*, and each treatment replicate had 6 wells, in a total of 18 or 6 seedlings, respectively. Continuous RLU from time zero (incubation time) up to 8 hours was detected using 1 mM of D-luciferin (Thermo Fisher cat #88293) as the substrate and a Veritas ™ microplate luminometer. Three RLU readings were made each hour.

### RNA extraction

One-week-old *Arabidopsis thaliana* WT (Col) or *RD29A::LUC* (Col) seedlings were treated with and without 200 µM ABA and 200 µM ABA + 1 µM FIPI for control DMSO only. One-week old *Arabidopsis thaliana* WT (Col) or *pld α1* (Col)-SALK_067533C or *pld δ* (Col) -SALK_023247C or *pld α1/δ* were also treated with 200 µM of ABA and for control DMSO only. One hundred mg of seedlings were collected after incubation for 4 hours and frozen in liquid nitrogen. Frozen seedlings were ground briefly to extract RNA, and 1 mL of TRIzol (Ambion Life technologies cat#15596026) was added to each sample. The samples were incubated at room temperature for 5 minutes, 200 µL of chloroform was added, and the samples were mixed. Tubes were left to stand for 3 minutes at room temperature and then centrifuged at 14,000 rpm for 15 minutes at room temperature. The aqueous phase was then collected, and 250 µL of isopropanol and 250 µL of a high salt solution (0.8 M sodium citrate and 1.2 M sodium chloride) were added. The samples were again incubated at room temperature for 10 minutes and then centrifuged at 14,000rpm for 10 minutes at room temperature. After the centrifuge step, the supernatant was discarded, and 1 mL of 70% ethanol was added to clean the pellet. The mixture was mixed by flicking the tube and centrifuged at 7,500 rpm for 5 minutes at room temperature. The pellet was air-dried for 1 hour at room temperature, and Kimwipes were used to collect excess ethanol from the tube. One hundred µL of nuclease-free water was used to dissolve the RNA, and RNA concentration was determined using a nanodrop spectrophotometer. RNA quality was also determined on a 2% agarose gel.

### Mutation confirmation by PCR

Homozygosity of the single mutant plants was confirmed using the following primers: FP CCAAAAGAGTTGTCGCTGAAG and RP CATTCTCTCACCACGTCATTG for *pld α1* and FP ATCCTACAGTGCAAATCGTGC and RP AGGAAAGGAAGTCAGGTGAGG for *pld δ. pld α1/δ* double knockout mutation was confirmed using the following primers: for *α1* mutation FP ATTAAGTGCAGGGCATTGATG and RP CAAGGCTGCAAAGTTTCTCTG and for *δ* mutation FP ATCCTACAGTGCAAATCGTGC and RP AGGAAAGGAAGTCAGGTGAGG.

### Semi-quantitative PCR

One µg/µL of RNA was treated with 1 µL DNAse (Promega cat# M6101) at 37°C for 30 minutes. One µL of DNAse stop was added to stop the reaction, and the mix was incubated at 65°C for 10 minutes. The treated RNA was then used to synthesize first-strand cDNA using (iScript cDNA synthesis kit Biorad cat# 1708890). One µL of iScript reverse transcriptase was added to 1 µg/µL treated RNA together with the reaction mix and 2 µL of 10 mM dNTP mix (NEB cat# N0447S) and adjusted to a 20 µL volume using nuclease-free water. Reaction settings for the synthesis were 5 minutes at 25°C, 20 minutes at 46°C, and 1 minute at 95°C. For amplification, the first strand sample was diluted by half, and 0.125 µL NEB Taq DNA polymerase (cat # M0273S) was added together with the 0.5 µL of 12 µM *LUC* and *ACTIN* forward and reverse primers each per reaction, 0.5 µL 10mM dNTP mix and 5 µL reaction mix. The amplification settings were: 2 minutes 96°C, 1-minute 94°C, 1 minute 64°C, and 1 minute 72°C, and the cycle was repeated from step 2. A total of 28 cycles were run. The products were separated using 2% agarose gel with ethidium bromide, and a Biorad gel analyzer chemidoc XRS+SYS was used to detect and image the bands using UV light illumination. ImageJ (Fiji) rectangle tool was used to determine the intensities of the bands. Primers used where *ACTIN 2* (AT3G18780) was the reference genes were FP CCCGCTATGTATGTCGC and RP AAGGTCAAGACGGAGGAT, and for *LUC*, the primers were FP GTTGTTGTTTTGGAGCACGGAAAGACG and RP CAACTCCTCCGCGCAACTTTTTCG.

### Quantitative PCR

RNA was treated with DNAse as above, and this was used in a 1-step qPCR reaction using iTaq Universal SYBR green one-step kit cat# 172-5150 with the QuantStudio·6 machine (Thermo Fisher) to quantify *RD29A* native expression. 500 ng/µL of treated RNA was mixed with 5 µL of the SYBR green reaction mix, 0.125 µL iScript reverse transcriptase, and 0.25 µL of 12 µM of both forward and reverse primers. The thermal cycling settings were 10 minutes at 50°C, 1 minute at 95°C, 15 seconds at 95°C, and 60 seconds at 60°C for 40 cycles. Data were analyzed using Quant Studio Real-Time PCR Software from Applied Biosystems. Primers used where *ACTIN 2* as reference gene were FP CCCGCTATGTATGTCGC, RP AAGGTCAAGACGGAGGAT, and *RD29A* native gene FP CACTCAACACACACCAGCAG and RP GGTGCATCGATCACTTCAGG.

### Stomata closure assay

Four to 6-week-old *Arabidopsis thaliana* WT (Col) or *pld α1* or *pld δ* leaves were harvested and placed in a stomata opening solution containing 5 mM KCl, 50 µM CaCl2, and 10 mM MES-Tris, pH 6.15 (Uraji et al., 2012b) for 2 hours to induce stomata opening. The leaves were then incubated with 100 µM of ABA only or 100 µM ABA with 0.6% butanol or, 0.1 µM FIPI or, 1 µM FIPI, or 100 µM PA only for 2 hours. The leaves were then shredded in a mini blender for 20 seconds. The mix was then filtered using a BD Falcon cell strainer 100 µm (cat#352360), and the epidermal layer pieces were placed on a Corning glass slide (cat#2948). 100 µL of the opening stomata solution was added to the pieces and covered with a Corning coverslip (cat# 2980). Stomata images were observed and captured using a Leica DMI microscope using a dry 40X objective lens. The stomata images were then analyzed using the ImageJ (Fiji) line tool, and the stomata aperture was determined to be the ratio between the width and length of the stomata.

### Microscope assay

One-week-old *Arabidopsis thaliana*, WT (Col), and *pldα1/δ* knockout mutants were incubated with Nitrobenzoxadiazole (NBD) labeled phospholipids; NBD-PC or NBD-PA on a Corning glass slide (cat#2948) and covered with Corning coverslip (cat# 2980). Images were taken on an Olympus SpinSR10 microscope using a 40x/1.4 oil objective lens. Photos were taken in a z-stack of 30 µm thickness with a z-step size of 1µm. Fluorescence was detected using a 464 nm excitation and 531 nm emission. Obtained images were then projected on the z-axis using ImageJ (Fiji) software.

## Results

### FIPI, a specific inhibitor of PLD, does not alter the promoter activity of *RD29A* carrying a single ABRE

**Figure 1:**
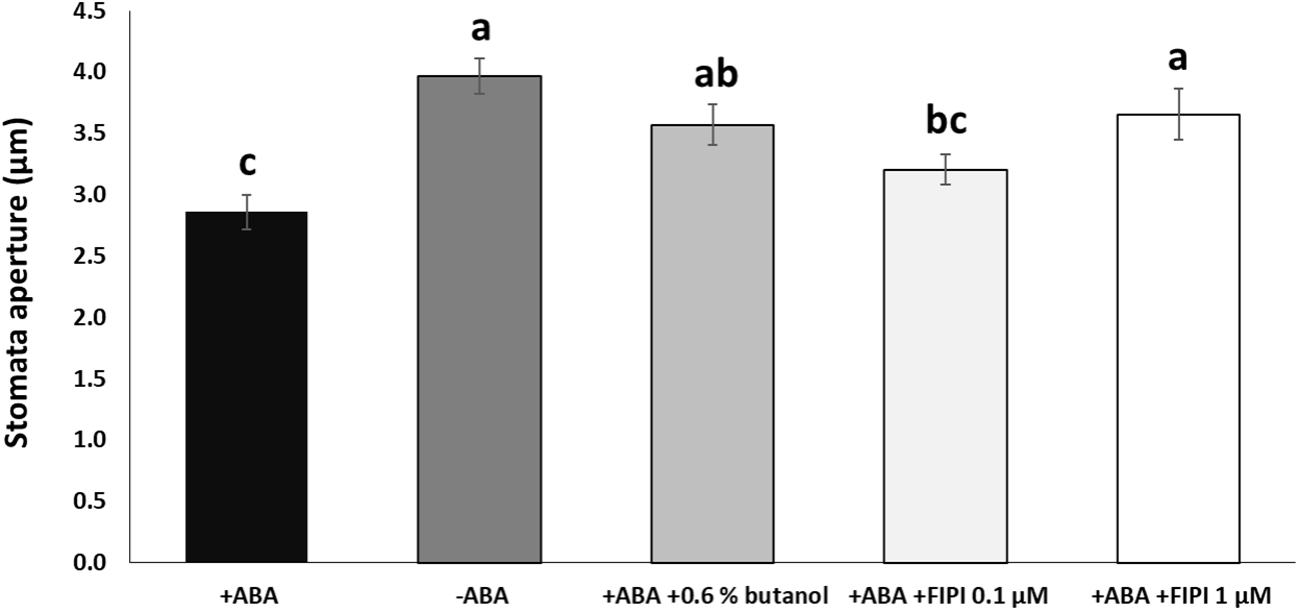
FIPI inhibits ABA-mediated stomatal closure. Stomata aperture sizes on wild-type leaves treated with and without ABA, ABA + 0.6% 1-butanol, ABA + 0.1 µM FIPI, or ABA + 1 µM FIPI for 2 hours. Each bar represents the means of 3 replicates of 60 stomata assayed with error bars representing standard error to the mean. Single letters **(a-c)** on top of each bar denote samples, among which statistically significant difference is not observed (*p>0.05* in Student’s t-test test).

To examine the effects of PA produced by the lipase activity of PLD on the *RD29A* promoter activity, we first conducted an *in vivo* enzyme inhibition assay. Although 1-butanol has been used as an *in vivo* PLD inhibitor in plants (Thiery et al., 2004; Motes et al., 2005; Johansson et al., 2014), a study in animals found that 1-butanol has deleterious effects on cells, including a reduction in phosphoinositide that is important in maintaining proper Golgi membrane structure (Su et al., 2009). Therefore, the inhibitory activity of 1-butanol is not necessarily related to the PLD activity. To this end, we used 5-Fluoro-2-indolyl des-chlorohalopemide (FIPI), which inhibits PLD activity more specifically than 1-butanol in cellular applications (Su et al., 2009; Stegner et al., 2013; Stricker et al., 2021). FIPI has been used to inhibit plant PLDs (Gao et al., 2013; Premkumar et al., 2019; Li et al., 2020). First, we confirmed stomatal closure inhibition by FIPI. In addition to 1 µM FIPI, ABA-mediated stomatal closure was inhibited significantly (**Figure 1**). A similar result was observed when the stomata were treated with ABA and 0.6% 1-butanol, conventionally used (**Figure 1**; **Supplementary S2**).

**Figure 2:**
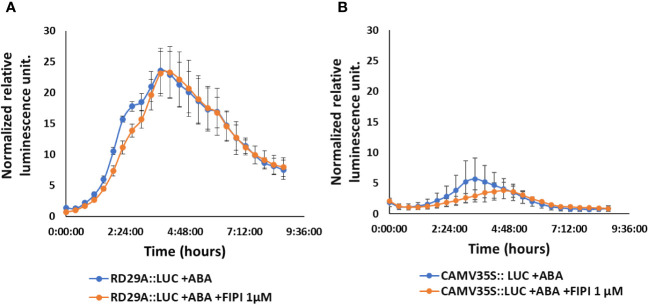
FIPI does not alter *RD29A* promoter activity in the *RD29A::LUC* plant. Normalized relative luminescence intensities are shown in *RD29A::LUC* **(A)** and *CAMV35S:: LUC* **(B)** seedlings treated with ABA or ABA + 1 µM FIPI. Relative intensities were determined and normalized at each point against control (with DMSO). The graph shows results from the means of 3 replicates with error bars from standard error to the mean.

We then treated the *RD29A::LUC* plants with either ABA only or with ABA and FIPI to determine the effect on the *RD29A* promoter activity. Adding 1 µM FIPI to ABA-treated plants did not alter the kinetics of luciferase activity (**Figure 2A**). For control, *CAMV35S::LUC* transgenic plants were used. The transgenic plants *CAMV35S::LUC* have a *CAMV35S* promoter fused to a firefly luciferase gene. The promoter does not contain the ABRE cis-element, and therefore, the plants act as a control for the luciferase reaction. The plants did not show significant effects on adding FIPI (**Figure 2B**), suggesting that FIPI does not affect the luciferase reaction.

**Figure 3:**
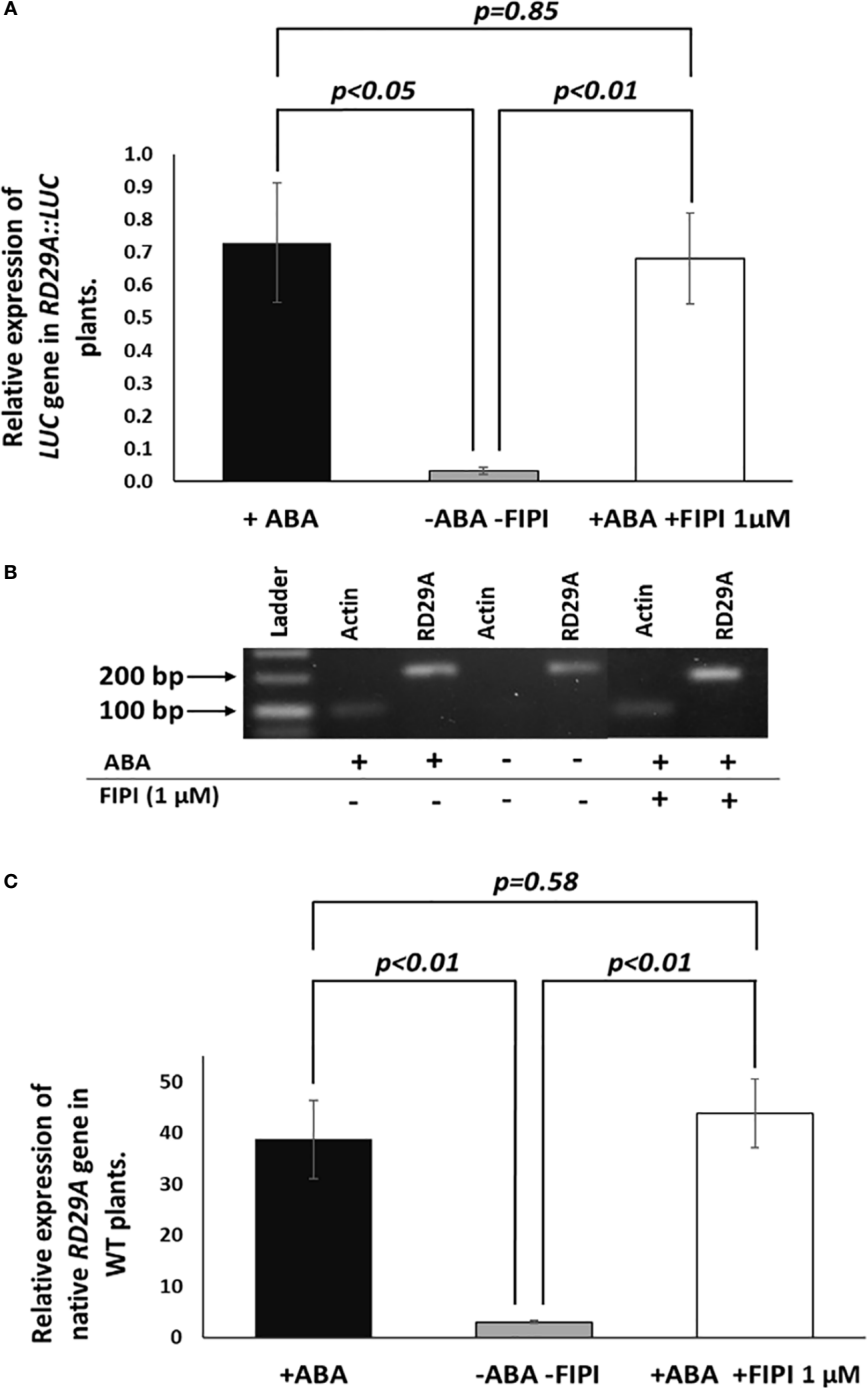
Expression and activation of *RD29A* are not alternated by FIPI. Plants were incubated for 4 hours with and without ABA or with ABA + 1 µM FIPI. In **(A)**, semi-quantitative PCR was conducted with the *RD29A::LUC* transgenic plants to determine the relative expression level of the luciferase gene against *ACTIN* mRNA. In **(B)**, a sample gel image for the semi-quantitative PCR is shown. In **(C)**, qPCR was conducted with WT plants to assess the relative gene expression level of *RD29A* against *ACTIN* mRNA. Each bar represents average expression levels of 3 replicates, with error bars representing standard error to the mean. A Student’s t-test was conducted between the +ABA and +ABA +1µM FIPI samples, and the *p values* are shown.

We then analyzed the accumulation of the luciferase (*LUC*) mRNA in the *RD29A::LUC* transgenic plants and the *RD29A* mRNA in wild-type (WT) plants (**Figure 3**). The results showed that 1 µM FIPI does not significantly affect the accumulation of the luciferase gene (**Figure 3A**) and *RD29A* in WT plants (**Figure 3B**). These results suggested that PLD enzyme activity does not alter the activity of the *RD29A* promoter that a single ABRE regulates.

### *PLD* knockout mutants *pldα1*, *pldδ, and pldα1/δ* do not affect the *RD29A* accumulation

There are 12 PLD enzymes in Arabidopsis, and 2 of these, namely PLDα1 and PLDδ, have been implicated in ABA signaling. ABA induces the expression of both PLDs, and plants deficient in these enzymes are impaired in ABA-mediated responses (Fan et al., 1997; Katagiri et al., 2001; Sang et al., 2001; Jia et al., 2013). Hence, we analyzed *pldα1*, *pldδ*, and *pldα1/δ* gene knockout mutants that are impaired in ABA-mediated stomatal closure. The mutant and WT plants were treated with ABA or DMSO, and the size of the stomatal apertures was compared. We found that the sizes of stomatal apertures in *pldα1* and *pldα1/δ* mutants were not significantly different with and without ABA treatment (**Supplementary Figure S3**). This result confirmed the previous reports that the *pldα1* and *pldα1/δ* plants, but not the *pldα1* plant, had reduced sensitivity against ABA in the stomatal closure (Uraji et al., 2012).

**Figure 4:**
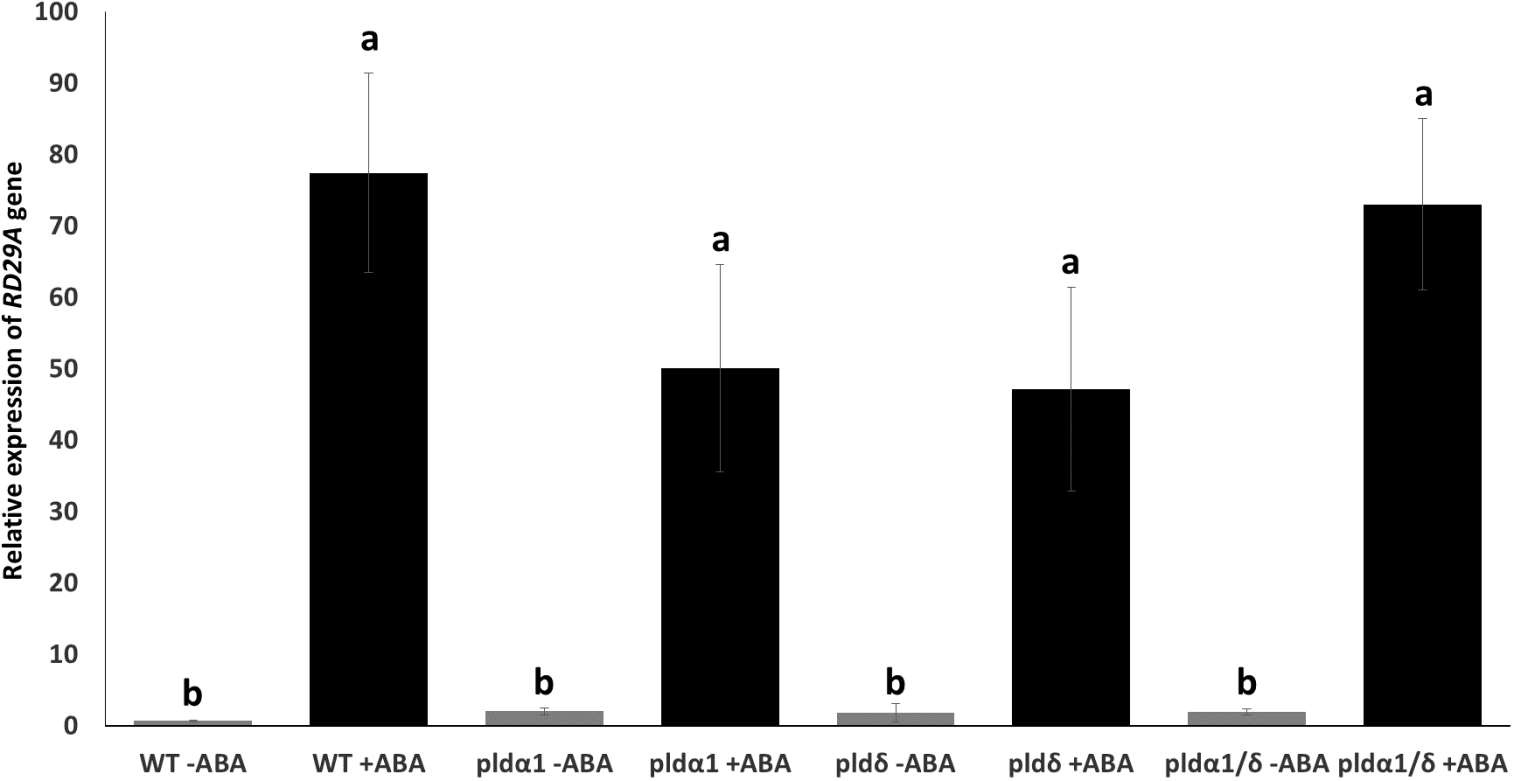
Mutant plants show relatively similar native *RD29A* expression to WT plants. Relative expression of *RD29A* in WT and mutant seedlings after incubation with and without ABA for 4 hours. qPCR was conducted with primers to amplify *RD29A* and *ACTIN* mRNAs. Relative expression levels of *RD29A* were calculated by normalizing with *ACTIN* mRNA. The analysis found that relative expression levels of *RD29A* tend to be higher in all mutant plants than WT plants without ABA, although none are significantly higher. When treated with ABA, no significant differences were observed between WT and the mutant plants. Each bar represents the mean expression levels of 3 replicates, with error bars representing standard error to the mean. One-way means ANOVA was conducted to determine significant differences among the samples and no significant differences were observed between plants within the same treatment. Single letters **(a, b)** on top of each bar denote groups, among which statistically significant difference is not observed (*p>0.05* in Student’s t-test).

We assumed these mutants might exhibit alternative expression of *RD29A* due to the lack of ABA-mediated enzymatic activity for PA production in the plants. We analyzed the expression of native *RD29A* by quantitative PCR (qPCR) without exposure to ABA and 4 hours after exposure to ABA. All the mutants, *pldα1*, *pldδ*, and *pldα1*/*pldδ*, did not show statistically significant changes in comparison to WT plants although they tended to show higher basal levels of the *RD29A* accumulation compared to WT plants without ABA treatment (**Figure 4A**). On treatment with ABA, all mutants showed similar *RD29A* expression levels as the WT, although the *RD29A* expression levels tended to be lower in the single mutants of *pldα1* and *pldδ* (**Figure 4B**) and higher in the double mutant. These results suggested that PLDs have little effect on the *RD29A* promoter activity.

### The addition of exogenous PA does not upregulate the promoter activity of *RD29A*

**Figure 5:**
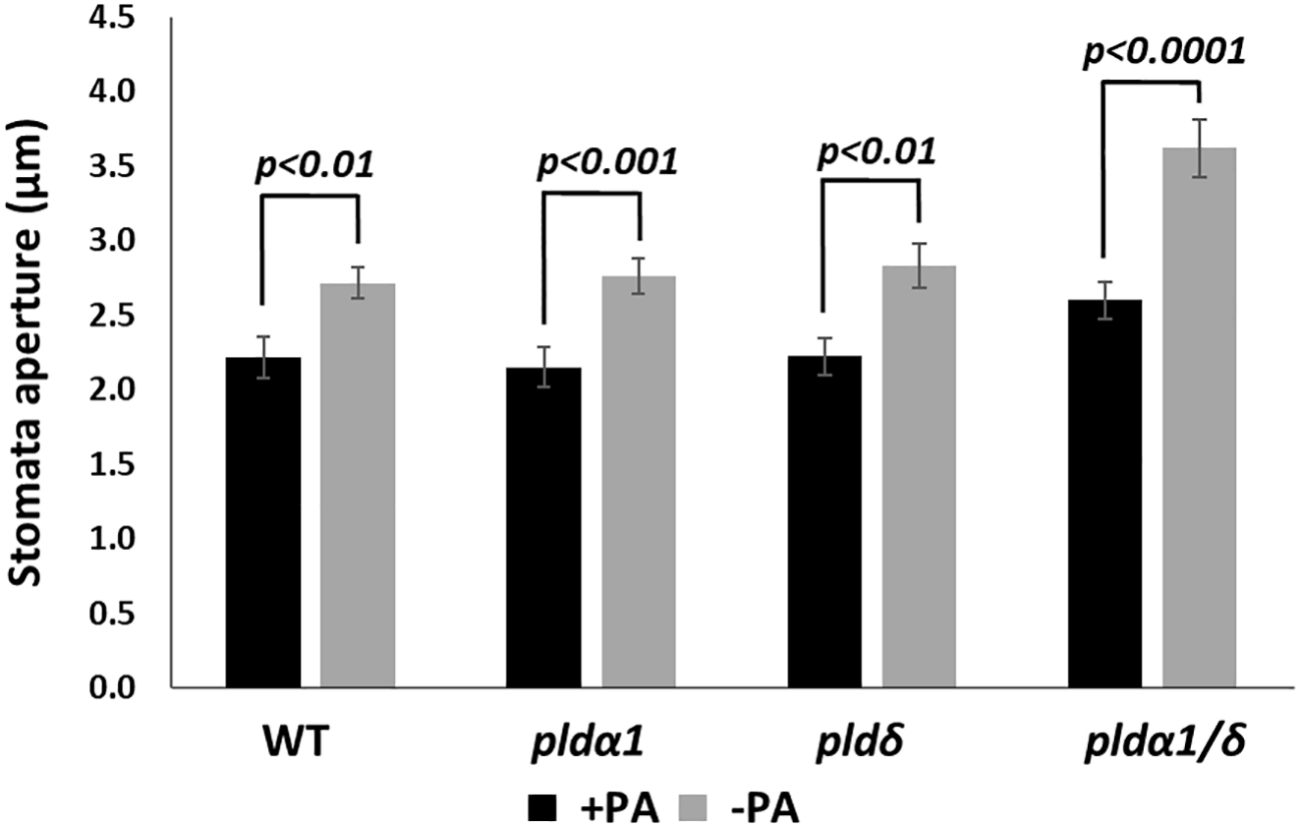
PA promotes stomatal closure in both WT and mutant plants. Stomata aperture sizes of WT, *pldα1*, *pldδ*, and *pldα1*/*pldδ* plants were treated with and without 100 µM PA for 2 hours. Each bar represents mean stomata sizes from 3 replicates of 60 stomata total assayed with error bars from standard error to the mean. A Student’s t-test was conducted between samples treated with PA and those not treated, and the respective *p values* in the Student’s t-test are shown.

To confirm that PA produced by the PLD enzymatic activity does not affect the *RD29A* promoter, we analyzed the effects of exogenously added PA in plants. First, we analyzed stomatal closure induced by adding PA (Uraji et al., 2012). Stomatal aperture sizes significantly reduced in WT, *pldα1*, *pldδ*, and *pldα1/δ* on the addition of PA, as previously reported (**Figure 5**). We also noted that the double mutant had larger stomatal apertures than other sample materials. This could be attributed to the difference in age of the sample material used., Overall, the trend of reduction of the aperture sizes by exogenous PA remained.

**Figure 6:**
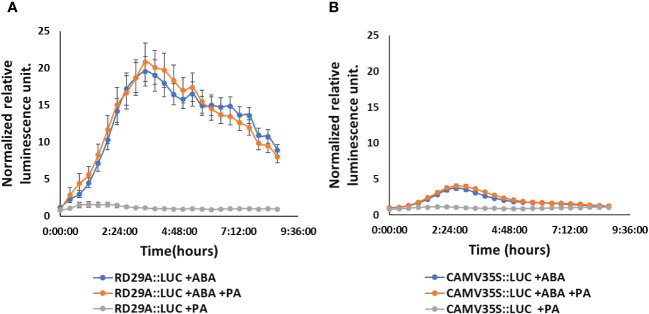
Exogenously added PA does not increase *RD29A* promoter activity in the *RD29A::LUC* plants. Normalized relative luminescence intensities in the *RD29A::LUC* plant **(A)** and *CAMV35S::LUC* plant **(B)** are shown. Seedlings were treated with ABA only or with ABA + 100 µM PA or with 100 µM PA only. Relative intensities were determined and normalized at each point against control (with DMSO). The graph shows results from the means of 3 replicates with error bars from standard error to the mean.

However, the treatment of PA on *RD29A::LUC* transgenic plants did not alter the activity of the promoter, including the treatment of the plants with a combination of PA and ABA (**Figure 6A**). These results confirmed that PA does not affect the *RD29A* promoter activity.

### Exogenously added PA is incorporated within the internal membranes in 2 minutes

We found that PA and PLDs have little effect on the *RD29A* promoter activity. We then questioned why PA produced by PLD affects stomatal closure but not gene expression during ABA signaling, although the core protein components of the signaling pathway are shared between them. A previous report suggested that ABI1 (PP2C) inhibition by PA during stomatal closure was caused by tethering of ABI1 in the plasma membrane (Zhang et al., 2004). Therefore, we hypothesized that the regulation of the ABA signaling pathway by PA would be limited in the plasma membrane where the SLAC1 channel protein is localized to regulate the stomatal closure (Hedrich, 2012). This would be why we did not see an effect of PA on ABA-mediated *RD29A* expression, which internally localized ABI1 would mediate.

We analyzed PA localizations using fluorescently labeled PA, Nitrobenzoxadiazole-PA (NBD-PA), to examine the hypothesis. The experiment aimed to determine whether exogenously added PA, first incorporated in the plasma membrane, remained in the plasma membrane, or incorporated into the internal membranes when ABA was added. If our hypothesis were true, we rationalized that the exogenously added PA would stay in the plasma membrane with and without ABA.

**Figure 7:**
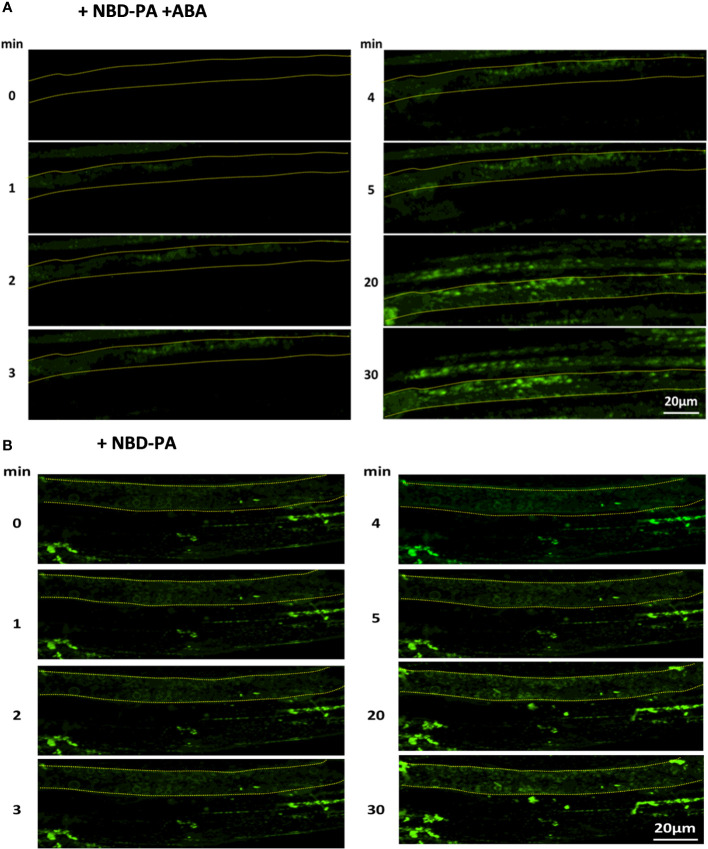
NBD-PA is internalized into the cells within minutes of exposure in Arabidopsis roots. NBD-PA fluorescence was detected in Arabidopsis roots of 1-week seedlings immediately after incubating with 50 μM NBD-PA with **(A)** and without **(B)** 100 μM ABA. Images were taken at different time points, as shown. A single cell is marked with a dashed-yellow line for clarification. Notice NBD signals are detected in the cytosolic area within 2 min with and without ABA.

Our fluorescence microscopy assay found that NBD-PA was rapidly incorporated into the internal membranes within 2 minutes after NBD-PA was exposed to WT seedlings with and without ABA (**Figure 7**).

This suggested that PA formed by PLDα1 or PLDδ would be rapidly internalized with and without ABA. This also indicated that PA created by PLD might play an internal role in the ABA signaling pathway, the same as the plasma membrane-localized PA.

We then analyzed the localization of exogenously added Nitrobenzoxadiazole-PC (NBD-PC) in seedlings of a *pldα1/δ* double knockout mutant. Because PA is produced from PC by the PLD activity, we rationalized that the *pldα1/δ* plant would show plasma membrane localization of NBD-PC signals. In contrast, the WT plant would show the internalized localization signals found with NBD-PA. We first analyzed whether the mutants, *pldα1*, *pldδ*, and *pldα1/δ*, were impaired in overall PLD activities in plants as previously reported (Zhang et al., 2004) (Ritchie & Gilroy, 1998). Namely, we analyzed PLD activity in the plants after treating them with fluorescently labeled PC (NBD-PC) and tracked its conversion to PA (NBD-PA) with thin-layer chromatography (TLC). The mutants showed reduced PA synthesis even without ABA treatment (**Supplementary Figure S4**). The *pldα1* mutant had reduced PA synthesis, although the activity was not significantly lower than the WT. The *pldδ* mutant had significantly reduced PA synthesis compared to the WT (**Supplementary Figure S5**). PA synthesis in the double knockout mutant of *pldα1/δ* was deficient even when treated with ABA (**Supplementary Figures S4** and **S5**). These results confirmed that the knockout mutants, especially the *pldα1/δ* double knockout, have reduced PLD activity in the plants.

**Figure 8:**
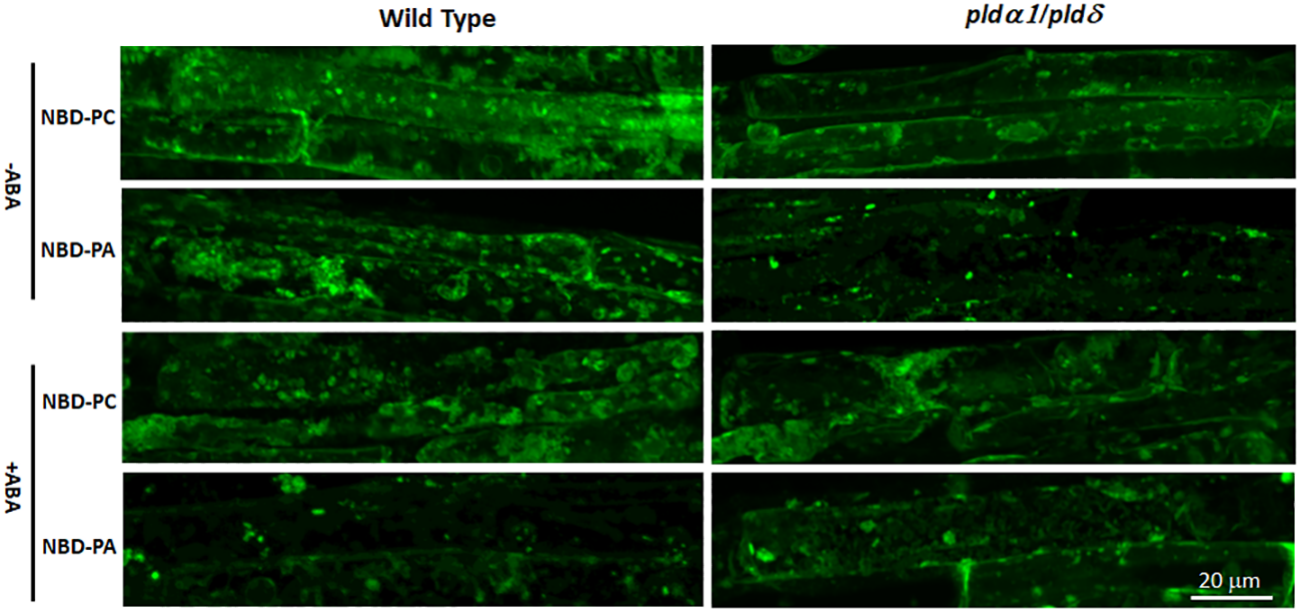
The distribution of NBD-PC and NBD-PA are indistinguishable between WT and *pldα1/δ* mutant plants. Arabidopsis roots were incubated with 50 μM NBD-PC or 50 μM NBD-PA with and without 100 μM ABA for 30 min. A single cell is marked with a dashed-yellow line for clarification. Notice that no significant difference is observed among the samples.

We then analyzed the NBD-fed plants with fluorescence microscopy and found that distributions of NBD-PC were not distinguishable between WT and *pldα1/δ* double knockout mutant plants (**Figure 8**). Similarly, distributions of NBD-PA were also not different between WT and *pldα1/δ* double knockout mutant plants. Typically, localization of NBD-PA and NBD-PC is not limited to the plasma membrane but dispersed in the internal components in WT and *pldα1/δ* double knockout mutant plants (**Figure 8**). This suggests that PLDα1 and PLDδ enzymes do not primarily affect the distribution of PC and PA within cells. We concluded that the hypothesis that the regulation of the ABA signaling pathway by PA would be limited to the plasma membrane was not valid.

## Discussion

*In vivo* enzyme inhibition of PLDs affects stomatal closure but not *RD29A* expression (**Figures 1**–**3**). We also found that knockout mutants *pldα1*, *pldδ*, and *pldα1/δ* do not show a statistically significant effect in the accumulation of *RD29A* (**Figure 4**). Moreover, adding exogenous PA affects stomatal closure but not the *RD29A* promoter activity (**Figures 5**, **6**). These results suggest that PA produced by PLDs and PLDs are not involved in the *RD29A* expression. These also suggest that previously observed upregulation of the *RD29A* expression in the *pldδ* knockout Arabidopsis (Distéfano et al., 2015) and in the *PLDα1* overexpressed tobacco (Peng et al., 2010) would not be due to alteration of the ABA signaling pathway. A recent study showed that the membrane lipid composition is altered in *pldα1* and *pldδ* knockout Arabidopsis plants (Zhou et al., 2022). In addition, the relationship between changes in membrane lipid composition and alterations in gene expression was previously identified in Arabidopsis plants (Szymanski et al., 2014), indicating that upregulation of the *RD29A* expression in the plants, *pldδ* knockout Arabidopsis (Distéfano et al., 2015) and the *PLDα1* overexpressed tobacco (Peng et al., 2010), may be due to changes in the membrane lipid composition. Furthermore, conditions not controlled in our study (i.e., oxygen and humidity) may affect the RD29A expression in the plants independently from ABA due to the existence of DRE in the promoter (Papdi et al. 2015; Zhou et al., 2022). These may also explain why we observed nearly 1.5-fold changes in the *RD29A* expression among the *pld* mutants, although the changes were statistically insignificant (**Figure 4**).

We also determined that exogenously added PA is incorporated into inner membranes within minutes in cells with and without ABA (**Figure 7**). Exogenously added phospholipids are first incorporated into the outer leaflet of the plasma membrane and transferred to the inner leaflet of the membrane by the enzyme flippase (Aminophospholipid ATPases) (Davis et al., 2020). Despite the requirement of an enzymatic reaction, exogenously added PA is detected in the cytosolic area of the cells within minutes. This suggests that PA formed in the plasma membrane can be spontaneously distributed into the internal membranes in the order of minutes. The double knockout mutant *pldα1/δ* displays a similar distribution of NBD-PC and NBD-PA compared to WT (**Figure 8**). These results do not support our hypothesis that the regulation of the ABA signaling pathway by PA would be limited to the plasma membrane. Instead, our finding supports the idea that PA inhibition on ABI1 does not require tethering in the plasma membrane (periphery of the cells) but can inhibit ABI1 within the cytosolic area of the cell (Umezawa et al., 2009); (Li et al., 2012). This can also explain how PA formed by PLDα1 binds and regulates SPHK1/2 localized in the vacuolar membrane (Pandit et al., 2018). Our analysis suggested that PA formed by PLDα1 in the plasma membrane can rapidly be transferred to the internal membranes, which may include a vacuolar membrane. Similarly, a previous study found that PLDα1 was also distributed within the cytosol in addition to the plasma membrane (Li et al., 2009), which agrees with the various distributions of NBD-PC and NBD-PA (**Figure 8**).

**Figure 9:**
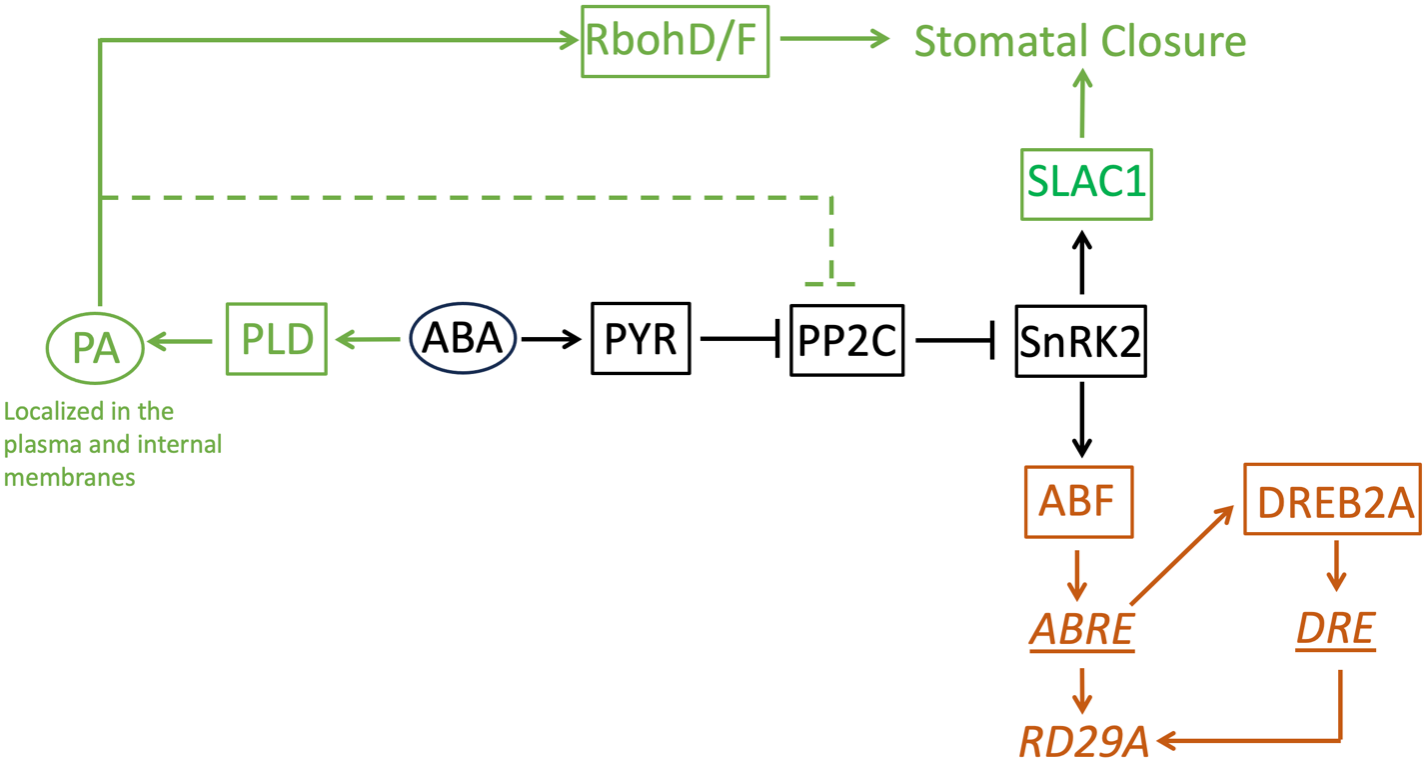
A model of the ABA-signaling network in stomatal closure and the *RD29A* expression. This study found phosphatidic acid is not involved in the *RD29A* expression. The core ABA-signaling component shared between stomatal closure and the *RD29A* expression is shown in black. The components involved in stomatal closure is shown in dark green. The components involved in the *RD29A* expression was shown in brown. Proteins are indicated with a square. Non-protein molecules are indicated with an oval. Gene expression regulatory elements are indicated with an underline. A gene is indicated with italic letters. The dashed line indicates the pathway to suppress selected members of the PP2C protein family that are involved in stomatal closure. RbohD/F: Respiratory burst oxidase homolog D/F, SLCA1: slow anion channel 1, PA: phosphatidic acid, PLD: phospholipase D, ABA: abscisic acid, PYR: pyrabactin resistance, PP2C: protein phosphatases 2C, SnRK2: SNF1-related protein kinase 2, ABF: ABRE-binding factor, DREB1A: dehydration-responsive element binding protein 2A, ABRE: ABA-responsive element, DRE: dehydration-responsive element, *RD29A*: Response-to-Dehydration 29A.

Then, why does PA affect stomatal closure but not the expression of the ABA marker gene, even though the signaling pathway is shared? Based on the results obtained in this study, we speculate on two factors. One is the presence of homologous proteins of PP2C that redundantly transduce the ABA signaling to the downstream protein, SnRK2. Genetic studies found that 4 homologous proteins, ABI1, ABI2, HAB1, and PP2CA, redundantly function as a suppressor of the OST1/SnRK2.6 protein that transduces a signal to close stomata and to up-regulate the ABRE promoter (Saez et al., 2006); (Nishimura et al., 2007); (Xue et al., 2008). Among the homologous proteins, only ABI1 is experimentally confirmed as a binding partner of PA (Zhang et al., 2004); Mishra et al., 2006). Although ABI2 is in the same clade as ABI1 in a phylogenetic analysis, HAB1 and PP2CA are in different clades, respectively (Xue et al., 2008), suggesting structural differences in the proteins. If we assume HAB1 and PP2CA do not bind to PA, it is reasonable that the effect of abolishing the production of PA in the ABA signaling pathway is minimal. Even though PA inhibits the function of ABI1 and ABI2, HAB1 and PP2CA can still function as an ABA signal transducer (**Figure 9**). Second, PA has dual roles in stomatal closure. It is found that PA produced by PLDα1 binds not only ABI1 but also RbohD/F, an NADPH oxidase enzyme (Zhang et al., 2009). The binding of PA to RbohD/F up-regulates ROS formation, which is required for stomatal closure but not necessary for the ABRE promoter activation (Zhang et al., 2009) (Song et al., 2014). When PA production by PLD is halted, production of ROS is also halted (Zhang et al., 2009). This makes stomatal closure impaired. On the other hand, even when PA production is stopped, the signal for the gene expression is still transduced (**Figure 9**). Previously, it was found that the expression of *RAB18* by the ABA exposure requires PA (Hallouin et al., 2002). This contradicts the finding that the expression of *RD29A* by ABA does not require PA in this study. However, the same research group found that the expression of *RAB18* also requires the activation of anion channels (Ghelis et al., 2000). This indicates the expression of *RAB18* requires an extra signaling pathway in addition to the core-signaling proteins PYR, PPC2, and SnRK2 (and ABF for the transcription). On the other hand, the expression of *RD29A* does not require the extra signaling pathway. Rather, it requires the binding of dehydration-responsive element binding protein 2A (DREB2A) that is expressed through the binding of ABF to the gene promoter of *DREB2A* to the dehydration-responsive element (DRE) in the *RD29A* promoter (Ndathe et al., 2022) (**Figure 9**). This allows the feedback to control the time scale and dynamic expression range in genes containing the single ABRE in the promoter.

In summary, this study solved a previously unsolved question about the involvement of PA in ABA-inducible gene expression. Our study found that PA does not activate the gene promoter containing an ABRE cis-element. Even when PA inhibits the ABI1 (PP2C) activity, the homologous protein, such as HAB1, accumulated in the same cell, would maintain the PP2C activity because HAB1 is PA insensitive. This situation would create two independent functional connections (i.e., PA-dependent and -independent phosphatase activity) from the same protein family in the ABA signaling network. As such, we cannot assume a protein family has the same connectivity to other proteins/molecules in the signaling network. Instead, we need to examine the connectivity by the subfamilies or individual proteins. It is still possible that PA alters other gene promoters independent from the ABA core signaling pathway, as was recently found in (Zhou et al., 2022). PA formed by PLD in the plasma membrane would be spontaneously transported to the cytosolic area of the cells within minutes. These new findings also should help clarify the role/connectivity of PA in the ABA signaling network.

## Data availability statement

The original contributions presented in the study are included in the article/**Supplementary Material**. Further inquiries can be directed to the corresponding author.

## Author contributions

RN: Investigation, Writing – original draft, Writing – review & editing. NK: Investigation, Methodology, Funding acquisition, Supervision, Writing – original draft, Writing – review & editing.
