# Supplementary material for: Phosphatidic acid produced by phospholipase Dα1 and Dδ is incorporated into the internal membranes but not involved in the gene expression of RD29A in the abscisic acid signaling network in Arabidopsis thaliana
Source: Front Plant Sci. 2024 Apr 12;15:1356699. doi: 10.3389/fpls.2024.1356699 (PMC11045897; doi:10.3389/fpls.2024.1356699)
Supplement: Supplementary file 1 [file DataSheet_1.pdf]

Supplemental Materials

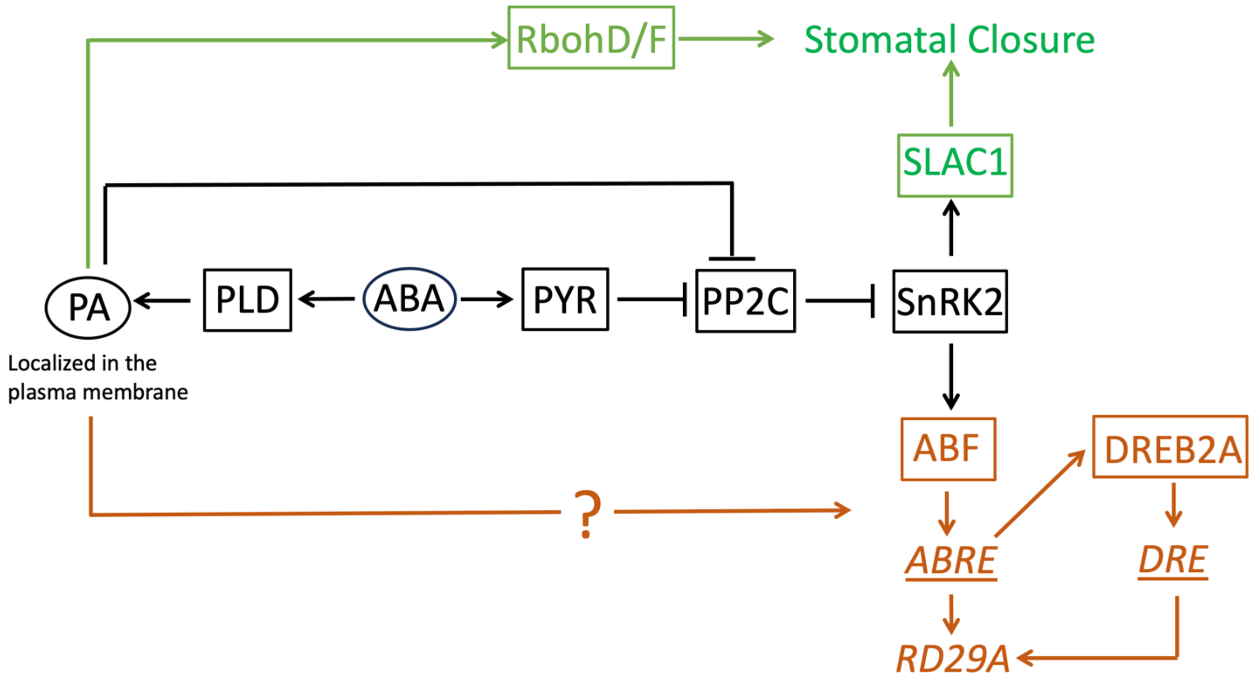

Figure S1. A previous model of the ABA-signaling network in stomatal closure and the *RD29A* gene expression. ABA-signaling components thought to be shared between stomatal closure and the *RD29A* gene expression are shown in black. Components involved in stomatal closure are shown in dark green. Components involved in the *RD29A* gene expression were shown in brown. Proteins are indicated with a square. Non-protein molecules are indicated with an oval. Gene expression regulatory elements are underlined. A gene is indicated with italic letters. A question mark indicates the suggested pathway. RbohD/F: Respiratory burst oxidase homolog D/F, SLCA1: slow anion channel 1, PA: phosphatidic acid, PLD: phospholipase D, ABA: abscisic acid, PYR: pyrabactin resistance, PP2C: protein phosphatases 2C, SnRK2: SNF1-related protein kinase 2, ABF: ABRE-binding factor, DREB1A: dehydration-responsive element binding protein 2A, ABRE: ABA-responsive element, DRE: dehydration-responsive element, RD29A: Response-to-Dehydration 29A.

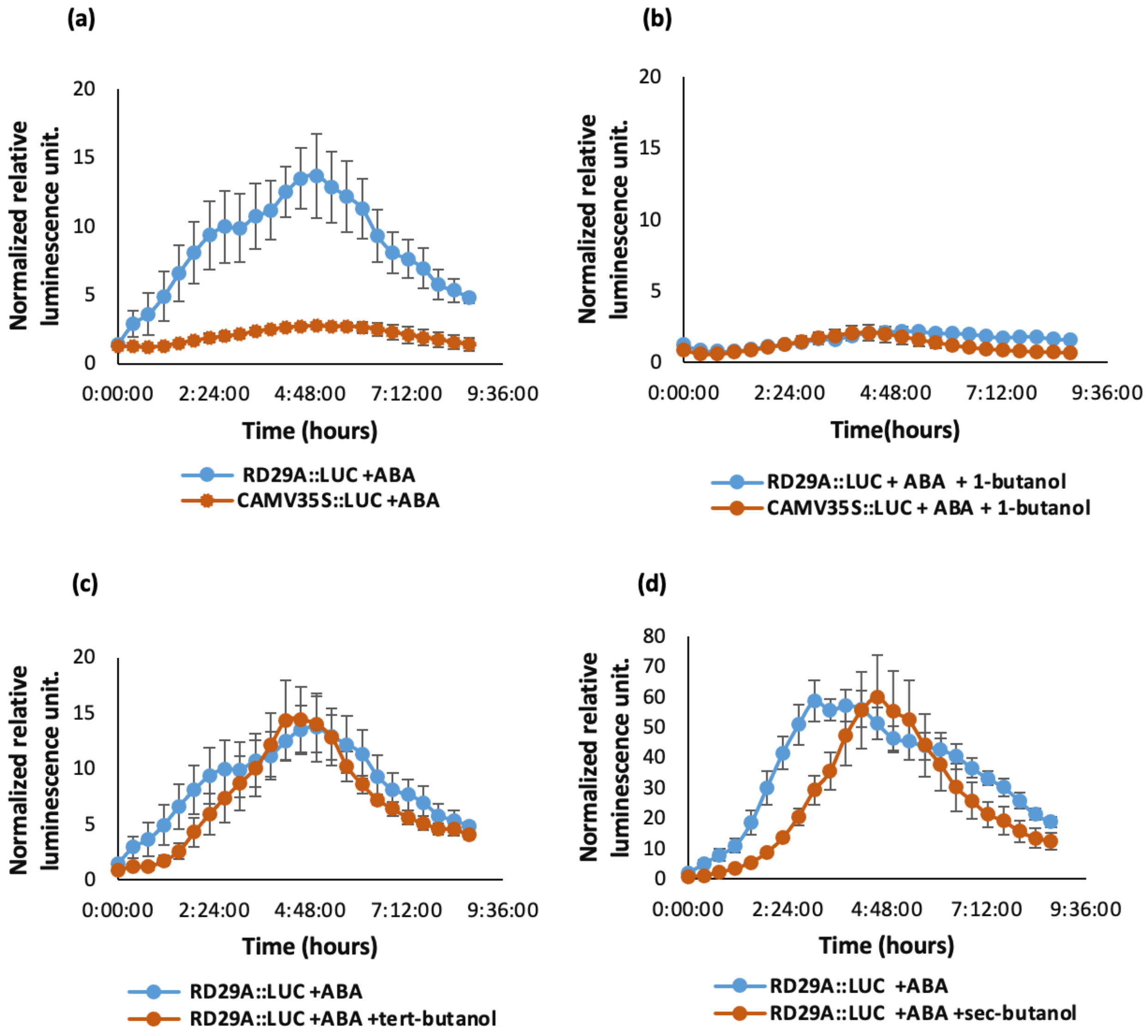

Figure S2. 1-butanol abrogates luciferase expression from the *RD29A* promoter but not from the *35S* promoter. Normalized relative luminescence intensities of *RD29A*::*LUC* and *CAMV35S::LUC* seedlings treated with ABA only in (a) or with ABA + 0.6 % 1-butanol in (b). The *RD29A::LUC* seedlings were also treated with ABA + 0.6% tert-butanol (c) and with ABA + 0.6% sec-butanol (d). Relative luminescence intensities were normalized against the control samples that were treated with DMSO. Each data point represents means of 3 replicates with error bars representing standard error from the mean.

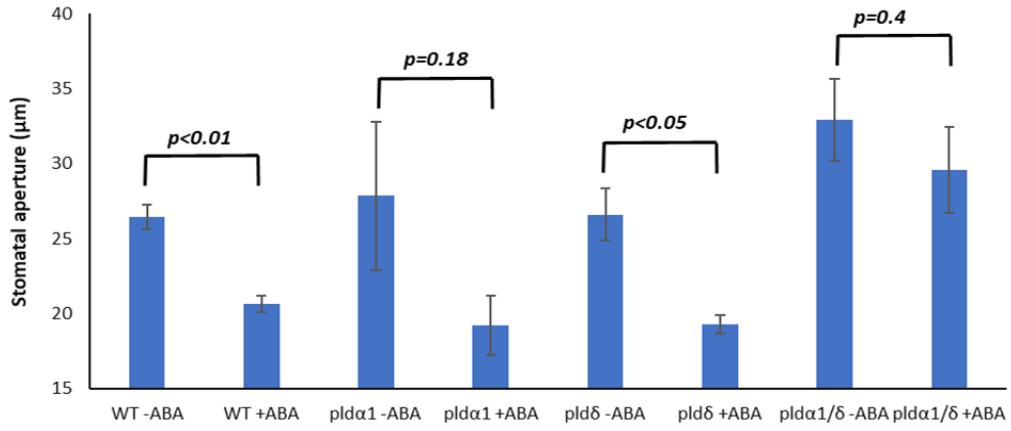

Figure S3. Mutant plants show reduced sensitivity to ABA in stomatal closure. Stomatal aperture sizes of WT, and *pldα1*, *pldδ* and *pldα1/δ* mutants when treated with 100 µM ABA or DMSO. Each bar except for *pldα1/δ* represents the average stomatal size from 3 replicates of 60 stomata assayed with error bars representing standard error to the mean. For *pldα1/δ,* the data is from one replicate of 20 stomata assayed and respective error bars representing standard error to the mean. *p values* from a Student's t-test between samples treated with and without ABA are shown.

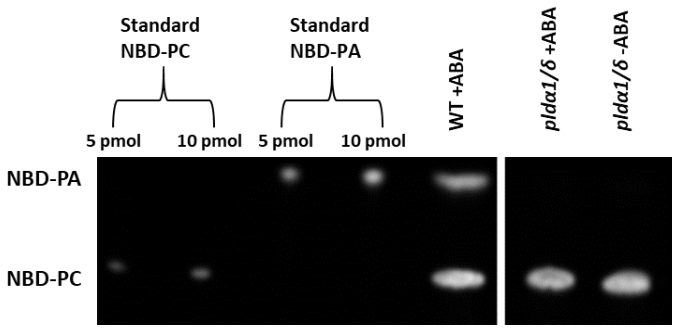

Figure S4. Determination of PLD activity in plants. A TLC analysis was conducted with lipid extracts from WT and *pldα1/δ* double knockout mutant plants. The plants were incubated with fluorescently labeled phospholipids PC (NBD-PC) for 2 hours and then with or without ABA for 30 min.

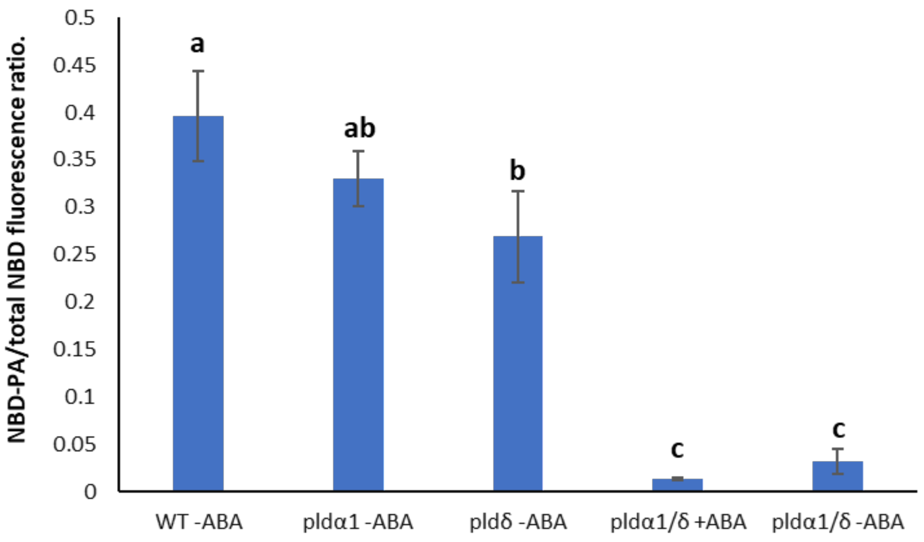

Figure S5. The *pldα1*, *pldδ,* and *pldα1/δ* mutant plants are impaired in PA synthesis from PC. PA synthesis was quantified in WT, and *pldα1*, *pldδ* and *pldα1/δ* mutants using fluorescently labeled lipid, NBD-PC. Seedlings were incubated with NBD-PC for $2^1/_2$ hours, and total lipids were extracted. The PLD activity was determined as a NBD-PA/ (NBD-PA + NBD-PC) ratio. The *pldα1/δ* knockout plants were incubated with and without ABA. The bars show means from three replicates of 20 seedlings each, and the error bars represent the standard error to the mean. Single letters (a, b, and c) on top of each bar denote samples, among which statistically significant difference is not observed (*p>0.05* in Student's t-test).
